# The Novel KIF1A Missense Variant (R169T) Strongly Reduces Microtubule Stimulated ATPase Activity and Is Associated With NESCAV Syndrome

**DOI:** 10.3389/fnins.2021.618098

**Published:** 2021-05-26

**Authors:** Cinthia Aguilera, Stefan Hümmer, Marc Masanas, Elisabeth Gabau, Miriam Guitart, A. Arockia Jeyaprakash, Miguel F. Segura, Anna Santamaria, Anna Ruiz

**Affiliations:** ^1^Genetics Laboratory, UDIAT-Centre Diagnòstic, Parc Taulí Hospital Universitari, Institut d’Investigació i Innovació Parc Taulí I3PT, Universitat Autònoma de Barcelona, Sabadell, Spain; ^2^Translational Molecular Pathology, Vall d’Hebron Research Institute (VHIR), Universitat Autònoma de Barcelona (UAB), Barcelona, Spain; ^3^Spanish Biomedical Research Network Centre in Oncology (CIBERONC), Madrid, Spain; ^4^Group of Translational Research in Child and Adolescent Cancer, Vall Hebron Research Institute (VHIR), Universitat Autònoma de Barcelona (UAB), Barcelona, Spain; ^5^Paediatric Unit, Parc Taulí Hospital Universitari, Institut d’Investigació i Innovació Parc Taulí I3PT, Universitat Autònoma de Barcelona, Sabadell, Spain; ^6^Wellcome Centre for Cell Biology, University of Edinburgh, Edinburgh, United Kingdom; ^7^Cell Cycle and Cancer Laboratory, Group of Biomedical Research in Urology, Vall Hebron Research Institute (VHIR), Universitat Autònoma de Barcelona (UAB), Barcelona, Spain

**Keywords:** NESCAV syndrome, KIF1A, kinesin, microtubule, motility, ATPase

## Abstract

KIF1A is a microtubule-dependent motor protein responsible for fast anterograde transport of synaptic vesicle precursors in neurons. Pathogenic variants in *KIF1A* have been associated with a wide spectrum of neurological disorders. Here, we report a patient presenting a severe neurodevelopmental disorder carrying a novel *de novo* missense variant p.Arg169Thr (R169T) in the KIF1A motor domain. The clinical features present in our patient match with those reported for NESCAV syndrome including severe developmental delay, spastic paraparesis, motor sensory neuropathy, bilateral optic nerve atrophy, progressive cerebellar atrophy, epilepsy, ataxia, and hypotonia. Here, we demonstrate that the microtubule-stimulated ATPase activity of the KIF1A is strongly reduced in the motor domain of the R169T variant. Supporting this, *in silico* structural modeling suggests that this variant impairs the interaction of the KIF1A motor domain with microtubules. The characterization of the molecular effect of the R169T variant on the KIF1A protein together with the presence of the typical clinical features indicates its causal pathogenic effect.

## Introduction

The kinesin superfamily of proteins (KIFs) are microtubule-dependent molecular motors that participate in the transport of membrane vesicles, organelles, protein complexes, and mRNAs along microtubules, thus playing important roles including mitosis, meiosis, and transport of cellular cargo such as axonal transport (Hirokawa et al., 1989). KIF1A belongs to the kinesin-3 family of kinesins and is a neuron-specific protein composed of an N-terminal motor domain (MD) (1–361 aa), which contains the ATPase activity and the microtubule-binding domains. These domains are followed by a short-strand “neck linker” (353–361 aa) and a neck coil region (NC) (365–397 aa) which are key regulatory elements for kinesin-3 motor dimerization and processive motility. The coiled-coil domains [CC1 (429–462 aa), CC2 (622–681 aa), and CC3 (801–822 aa)] together with the forkhead-associated domain (516–572 aa) are involved in the autoregulation of the motor activity and in the dimerization of the protein. The pleckstrin homology domain (1676–1774 aa) is implicated in cargo binding (Klopfenstein et al., 2002; Hammond et al., 2009; Huo et al., 2012). KIF1A exists in a dimeric inactive state that is maintained by autoinhibitory mechanisms and are activated upon cargo binding, enabling KIF1A to transport synaptic vesicle precursors along microtubules (Okada et al., 1995; Lee et al., 2003; Hammond et al., 2009).

Pathogenic variants in *KIF1A* have been linked to four clinical different disorders: (i) neuropathy, hereditary sensory, type IIC (HSNIIC, MIM # 614213); (ii) Spastic Paraplegia 30, autosomal recessive (SPG30, MIM # 610357); (iii) Spastic Paraplegia 30, autosomal dominant (SPG30, MIM # 610357); and iv) neurodegeneration and spasticity with or without cerebellar atrophy or cortical visual impairment (NESCAV syndrome, NESCAVS, MIM # 614255, formerly known as mental retardation autosomal dominant 9).

The NESCAV syndrome always occurs as a consequence of *de novo* missense variants located in the motor domain. The clinical features include moderate to severe intellectual disability (ID), language and motor delay, hypotonia, spastic paraparesis, hyperreflexia, postnatal microcephaly, and peripheral neuropathy. MRI shows optic nerve atrophy and varying degrees of brain atrophy, the cerebellum being the most severely affected. It is thought that the variants leading to this clinical presentation disrupt the motility of the KIF1A protein along the microtubules (Esmaeeli Nieh et al., 2015; Lee et al., 2015).

Functional studies performed on hippocampal neurons show a significant reduced distal localization of mutant motor domain constructs harboring the *de novo* missense variants T99M, A202P, S215, R216P, and E253K compared with WT motor domain in neurites (Lee et al., 2015). In addition, experiments performed in SH-SY5Y cells demonstrated that the *de novo* missense variants C92R, D248E, and P305L caused an accumulation of mutated protein preferentially in the cell body instead to the tips of neurites, where wild-type KIF1A is located (Kaur et al., 2020). Moreover, *in vitro* gliding assays showed loss of motility for *de novo* missense C92R, T99M, R216C, D248E E253K, and P305L mutant motor domains (Esmaeeli Nieh et al., 2015; Kaur et al., 2020). The equimolar mix of WT and E253K showed an 8-fold reduction in microtubule gliding velocities, suggesting a dominant negative effect (Esmaeeli Nieh et al., 2015).

Interestingly, alternative missense variants in the motor domain have been demonstrated to be responsible for the dominant and recessive spastic paraplegia phenotype not associated with ID. It has been proposed that variants causing this phenotype may have a milder effect on the motility of KIF1A along microtubules, resulting in a lower impairment of synaptic vesicle anterograde transport and possibly only affecting long axons. Alternatively, it has been suggested that a redundancy in the expression of kinesins in affected neuronal populations can compensate KIF1A variants with milder effect (Hirokawa and Tanaka, 2015; Pennings et al., 2020). However, a recent study has demonstrated that some spastic paraplegia pathogenic variants (V8M, A255V, and R350G) confer a gain-of-function activity to the KIFA protein resulting in a hyperactivation of the kinesin motor domain (Chiba et al., 2019). Thus, the reason why different variants cause different types of diseases still remains unclear.

Here, we present the identification of a patient affected by a severe neurodevelopment disorder who is a carrier of a novel *de novo* missense variant in the motor domain of KIF1A. The variant p.Arg169Thr (R169T) has not been described before in other patients with ID or spastic paraplegia and is not present in the gnomAD database of control individuals. *In silico* prediction tools suggest a deleterious effect of the variant on the protein. *In vitro* analysis of the R169T variant revealed a strong inhibition of microtubule-stimulated ATPase activity. Moreover, structure-based modeling predicts that R169T variant may directly interfere with the interaction of KIF1A motor domain with microtubules.

## Materials and Methods

The study has been approved by the institutional Ethics Committee of Institut d’Investigació i Innovació Parc Taulí I3PT (CEIC 2019/514), and the corresponding informed consent has been obtained from the parents.

### Whole-Exome Sequencing and Variant Detection

Trio WES of the patient and his parents was performed using the SureSelect Human All Exon V5+UTR kit (Agilent Technologies, Santa Clara, United States). Sequencing was performed at the National Center of Genomic Analysis (CNAG-CRG, Barcelona, Spain) on an Illumina HiSeq 2000 platform (Illumina, San Diego, CA, United States), producing 2 × 100 nucleotides paired end reads. Raw reads were mapped to the human reference genome (hg19) using the BWA aligner (Li and Durbin, 2010) and subsequently processed using the GATK pipeline in order to remove PCR duplicates and perform base quality score recalibration. Variant discovery was performed using the Haplotype Caller tool and following the best practices for exome sequencing variant discovery of GATK (DePristo et al., 2011). Only variants with a called genotype and genotype quality ≥ 20 in all the trio members were considered.

Finally, all variants were annotated using ANNOVAR (Wang et al., 2010). All exome variants were checked against a *de novo* model of inheritance, and variants were filtered for a population allele frequency of less than 1/10,000 in the gnomAD database (Karczewski et al., 2020) and a predicted deleterious effect on the protein (nonsense, frameshift, splicing, and missense variants were prioritized). The impact of missense variants was assessed using several in silico tools including PolyPhen-2 (Adzhubei et al., 2010), Mutation assessor (Reva et al., 2007), PROVEAN (Choi and Chan, 2015), MutPred (Pejaver et al., 2017), CADD (Rentzsch et al., 2019), Primate AI (Sundaram et al., 2018) and REVEL (Ioannidis et al., 2016).

### Plasmids

pMA-RQ KIF1A motor domain WT (3–362 aa) and R169T were obtained from GeneArt Gene synthesis service (Thermo Fisher Scientific, Massachusetts, United States). pcDNA3.1(+) KIF1A-C-Myc was obtained from GenScript Biotech (Clone ID OHu22637). To obtain the R169T variant, site-directed mutagenesis was performed using the QuikChange II Site-directed mutagenesis kit (Agilent Technologies, Santa Clara, United States) following the manufacturer’s instructions. Mutagenesis primers are listed in Supplementary Table 1.

### Cloning and Production of Recombinant KIF1A R169T and WT Motor Domains

The motor domains of KIF1A WT and R169T (3–362 aa, NP_001230937.1) were obtained from the GeneArt Gene synthesis service cloned in the pMA-RQ plasmid. The WT and R169T motor domains were subcloned into a pQE-80L expression vector with a hexa-histidine tag, kindly provided by Dr. Thomas U. Mayer. The constructs were transformed in the BL21-Codon Plus RIL cells (Agilent technologies), and protein expression was induced with 1 mM IPTG (isopropyl β-d-1-thiogalactopyranoside), at 18°C overnight. The expression and solubility of WT and R169T motor domains were tested by western blot, staining the PVDF (polyvinylidene difluoride) membrane with Naphthol Blue and using a penta-his antibody (included in QIAexpress Ni-NTA Fast Start kit). Protein purification was performed with the QIAexpress Ni-NTA Fast Start kit (Qiagen, Hilden, Germany) following the manufacturer’s instructions. After purification, a western blot and Naphthol Blue staining of the PVDF membrane was carried out in order to confirm the presence of the purified WT and R169T motor domains. Finally, the recombinant proteins were dialyzed overnight at 4°C against a buffer containing 20 mM Tris–HCl pH 7.4, 300 mM NaCl, 10 mM β-mercaptoethanol, 10% de glycerol, and 150 μM ATP.

### Overexpression of KIF1A in the SH-SY5Y Cell Line

The human neuroblastoma cancer cell line SH-SY5Y (bone marrow) was purchased from the American Type Culture Collection (ATCC, CRL-2266). Cells were grown at 37°C under a humidified atmosphere (5% CO_2_) conditions in Iscove’s modified Dulbecco’s Medium (Thermo Fisher Scientific) supplemented with 10% fetal bovine serum (Sigma-Aldrich) and 1% of Insulin-Transferrin-Selenium Supplement. A total of 520,000 cells/well were transfected with pcDNA3.1 (+) empty, pcDNA3.1 (+) KIF1A-C-myc WT, and KIF1A-C-myc R169T using Lipofectamine 2000 (Thermo Fisher Scientific), following the manufacturer’s instructions. Cells were homogenized in RIPA lysis buffer (Thermo Fisher Scientific), and total protein content was quantified using the DC Protein Assay Kit (Bio-Rad). Thirty micrograms of protein was separated on 4–12% SDS-PAGE gel and transferred onto a PVDF membrane (Sigma-Aldrich). The membrane was blocked in 5% BSA (Sigma-Aldrich) and incubated with the Anti-KIF1A antibody (diluted 1:1,000, Abcam ab180153).

### *In vitro* ATPase Assay

The *in vitro* ATPase assay was performed with the Kinesin ATPase Endpoint Biochem kit (Cytoskeleton Inc., Denver, United States). The concentration of KIF1A WT and R169T motor domains was adjusted to 0.5 μg/μl, then a dilution to a final concentration of 0.03 μg/μl was performed using Kinesin Reaction Buffer. To test the ATPase activity, the indicated amounts of the diluted WT and R169T motor of domains, ranging from 0.0078 to 0.24 μg, were added to each reaction. For the basal ATPase activity, Kinesin Reaction Buffer without microtubules was added to a final volume of 30 μl. For the microtubule-stimulated ATPase activity, 10 μl of taxol-stabilized microtubules (0.2 mg/ml) and Kinesin Reaction Buffer to a final volume of 30 μl were added. The ATPase reaction was started by adding 10 μl of 100 mM ATP PIPES pH 7.0 (Sigma Aldrich, Missouri, United States) and incubating 5 min at room temperature. To terminate the reaction, 70 μl of CytoPhos reagent was added and incubated for 10 min at room temperature. Absorbance at 650 nm was measured by Epoch Microplate Spectrophotometer (BioTeK, Agilent Technologies). In order to calculate the activity, the absorbance of the reaction with microtubules was subtracted from the absorbance of the reaction without microtubules. ATPase activity was tested in triplicate, and the estimation of the amount of inorganic phosphate (Pi) released through the ATPase activity was determined by using a Pi standard curve. For experiments with increasing concentrations of microtubule form (0–4.375 μM), 0.015 μg/μl of WT motor domain and 0.06 μg/μl of R169T were used. Titration curves were performed in triplicate, and activity (nmol min^–1^) was blotted against microtubule concentration (μM). Double reciprocal analysis of the ATPase activity vs. microtubule concentration was performed and data were fitted by linear regression. K_*m*_ and V_max_ were determined as intersections with the $\text{X}=\frac{-1}{{\text{K}}_{\text{m}}}$ and $\text{Y}=\frac{1}{{\text{V}}_{\text{max}}}$ axis, respectively, according to the Lineweaver Burk equation ($\frac{1}{\text{V}}=\frac{{\text{K}}_{\text{m}}}{{\text{V}}_{\text{max}}}\,\text{⋅}\,\frac{1}{\text{[S]}}+\frac{1}{{\text{V}}_{\text{max}}}$). K_cat_ was determined by ${\text{K}}_{\text{cat}}=\frac{{\text{V}}_{\text{max}}}{\left[\mathrm{Total}\,\mathrm{enzyme}\,\mathrm{concentration}\right]}$.

### Structural Modeling of the KIF1A R169T Missense Variant

The R169T mutation was modeled in the published KIF1A motor domain crystal structure (2HFX) using PyMOL, a molecular graphics program (The PyMOL Molecular Graphics System, Version 1.2r3pre, Schrödinger, LLC).

## Results

### Clinical Description of the Patient

**FIGURE 1:**
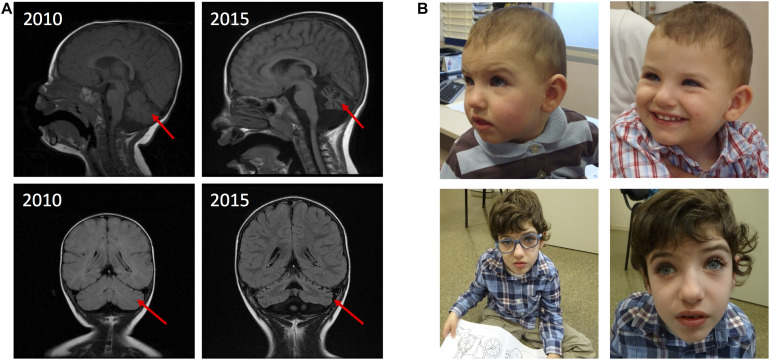
MRI findings and pictures of the patient. **(A)** MRI showing progressive cerebellar atrophy. **(B)** Patient at 1 year of age (top left), at 1 year and 9 months of age (top right) and at 8 years of age (in the bottom).

The proband is a 10-year-old boy born at term from non-consanguineous parents. Birth weight was 2,750 g. No perinatal problems were reported, except for gastroesophageal reflux. At the age of 10 months, he presented global development delay, microcephaly, hypotonia, feeding difficulties, stereotypies, and an abnormal electroencephalogram (EEG). MRI showed a delayed myelination. Genetic analysis included Angelman syndrome testing (15q11-q13 methylation pattern and *UBE3A* sequencing), karyotype, fragile X syndrome testing, subtelomeric MLPA (SALSA MLPA P070, MRC Holland), and 60K array Comparative Genomic Hybridization (aCGH) (Agilent Technologies) with normal results. At the age of 5 years, the patient developed epilepsy and MRI scans showed partial bilateral optic atrophy and progressive cerebellar atrophy (Figure 1A). Currently, the patient presents severe ID, speech impairment, postnatal progressive microcephaly (-2SD), hypotonia, partial occipital epilepsy, unsteady gait, feeding problems (dysphagia), hyperreflexia, ataxia, peripheral motor sensory neuropathy, and short stature. The behavioral phenotype includes apparent happy demeanor, easily excitable personality, and stereotypies (Figure 1B and Table 1).

**TABLE 1:** Clinical features of the patient according to what is described for NESCAV syndrome.

Clinical features associated with NESCAV syndrome	Patient with c.506G > C, p.Arg169Thr *de novo* missense variant
Moderate to severe developmental delay/ID	+
Severe motor delay	+
Ataxia	+
Peripheral neuropathy	+
Axial hypotonia	+
Spastic paraparesis	+
Hyperreflexia	+
Microcephaly	+
Optic nerve atrophy	+
Abnormal eye movements	+
Mild to moderate language delay	+
Epilepsy	+
Epileptic abnormalities on EEG	+
Metatarsus adductus	+
**MRI**	
Brain atrophy	–
Diminished cerebral white matter	–
Severe and progressive cerebellar atrophy	+

**+, present;* –, *not present.**

### Identification of a *de novo* R169T Variant in the *KIF1A* Gene

Trio-WES analysis identified a total of three variants after filtering the exome data according to a *de novo* model of inheritance, a population allele frequency of less than 1/10,000, and a predicted deleterious effect on the protein (Supplementary Table 2). A novel *de novo* missense variant, c.506G > C, p.Arg169Thr (R169T), in the *KIF1A* gene (NM_001244008.2) was selected as the best candidate. *De novo* variants in the *KIF1A* gene are associated with NESCAV syndrome, whose clinical features match with those described in our patient (Table 1; Esmaeeli Nieh et al., 2015; Lee et al., 2015). The variant is not present in the gnomAD database. Several bioinformatic tools were used to predict the functional effect of the variant on the protein, all of them suggesting a strong damaging effect (Table 2). The variant was classified as likely pathogenic following the ACMG guidelines (Richards et al., 2015). The variant has not been described before and was submitted to the ClinVar database (ref. VCV000666256).

**TABLE 2:** *In silico* analysis of the missense variant p.Arg169Thr.

*In silico* tool	Prediction	Score
PROVEAN	Deleterious	–5.36
PolyPhen-2	Probably damaging	0.991
Mutation assessor	Medium	3.29
MutPred	Deleterious	0.833
CADD	Deleterious	26.2
PrimateAI	Damaging	0.8787
REVEL	Pathogenic	0.8569

### Functional Analysis of the R169T Variant

#### The KIF1A R169T Variant Does Not Alter the Protein Stability

**FIGURE 2:**
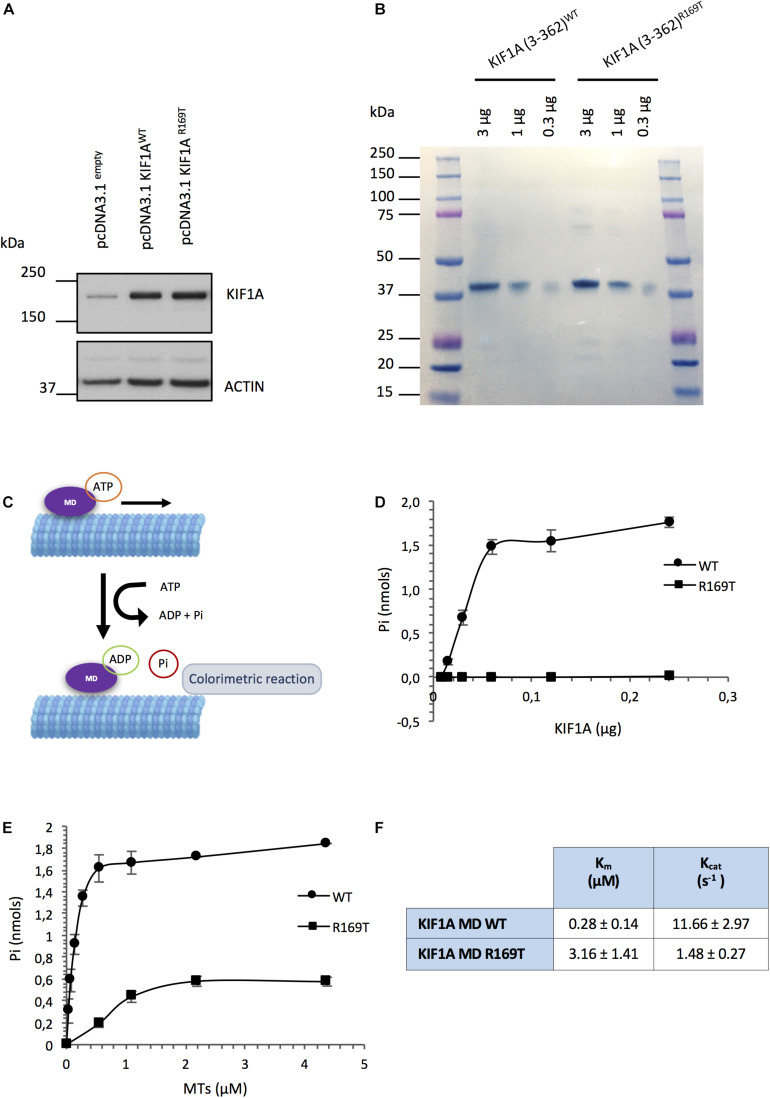
Functional assays carried out with the R169T variant. **(A)** Overexpression of full-length KIF1A WT and R169T in SH-SY5Y neuroblastoma cells. Cell lysates were harvested 96 h post-transfection. Thirty micrograms of protein corresponding to SH-SY5Y cells transfected with pcDNA3.1^*empty*^, pcDNA3.1 KIF1A^*WT*^, and pcDNA3.1 KIF1A^*R16*9T^ vectors loaded. Actin was used as a loading control. **(B)** Naphthol Blue staining of the PDVF membrane showing that the same amount of protein corresponding to WT and R169T has been used for the ATPase assay. **(C)** ATPase activity assay diagram. **(D)** Microtubule-stimulated ATPase activity of the WT and R169T motor domains. A range of 0.0078–0.24 μg of recombinant protein was used in the assay. To start the ATPase reaction, 10 μl of taxol-stabilized microtubules (0.2 mg/ml) and 10 μl of ATP 100 mM ATP PIPES pH 7.0 were added to each well and incubated 5 min at room temperature. The readout at 650 nm was performed after a 10-min incubation of the reactions with 70 μl of CytoPhos termination reagent. **(E)** ATPase activity in the presence of increasing concentrations of microtubules. A range of 0–4.375 μM of microtubules was used. Data were fitted by linear regression. K_*m*_ and V_max_ were determined according to the Lineweaver Burk equation. K_cat_ was determined by K_*cat*_ = V_max_/[total enzyme concentration]. Pi, inorganic phosphate; MTs, microtubules. **(F)** K_*m*_ and K_cat_ values of KIF1A motor domain (MD) WT and R169T.

Missense variants can affect the protein folding as well as protein stability as a consequence of changes in the physiochemical characteristics or conformational constraints. It has been reported that over 80% of all disease-associated missense variants are amino acid substitutions that affect protein stability (Wang and Moult, 2001; Zhang et al., 2012). In the absence of a cell line model carrying the variant R169T, we ectopically expressed the WT and mutant KIF1A forms in SH-SY5Y neuroblastoma cells and determined protein stability by western blot. The results show that the R169T and WT constructs are expressed at similar levels and had the same electrophoretic mobility, thereby indicating that the ectopically expressed variant R169T does not affect the protein stability (Figure 2A).

#### R169T Missense Variant Impairs KIF1A ATPase Activity

In order to assess the effect of the variant on the functionality of the KIF1A motor domain, we purified the wild-type and mutant recombinant KIF1A motor-domain proteins (3–362 aa) (Supplementary Figure 1) and performed an ATPase assay. The inorganic phosphate (Pi) generated at different concentrations of purified WT and R169T motor domains was measured following induction of microtubule-stimulated ATPase activity. We found that while the release of Pi increased in function of the concentration of the WT motor domain, there was a strong reduction in the amount of Pi generated by the R169T motor domain. The microtubule-stimulated ATPase rate of the R169T motor domain was about 180-fold lower than the motor domain of WT KIF1A (8.3 nmol of ATP hydrolyzed/min/mg vs. 1466.6 nmol of ATP hydrolyzed/min/mg; Student’s *t*-test, *p*-value 0.0095). Thus, the ATPase activity of the KIF1A motor domain is severely affected by the variant R169T (Figures 2B–D).

To test if this defect is caused by impaired stimulation of the ATPase activity by microtubules, we determined the ATPase activity in KIF1A WT and R169T with different amounts of microtubules. In the case of the R169T mutant form, ATPase activity can only be achieved in the presence of high concentrations of microtubules (i.e., 2.18 μM) (Figure 2E). Determination of the K_*m*_ and K_cat_ values revealed an 11-fold increase in K_*m*_ (0.28 ± 0.14 μM vs. 3.16 ± 1.41 μM) and 8-fold decrease in K_cat_ (11.66 ± 2.97 s^–1^ vs. 1.48 ± 0.27 s^–1^) of the R169T mutant compared with the WT motor domain (Figures 2E,F). Therefore, these results indicate that the variant R169T strongly affects the microtubule-stimulated ATPase activity, which results in a reduction in the maximal ATPase rate.

### Structural Modeling of the KIF1A R169T Missense Variant

**FIGURE 3:**
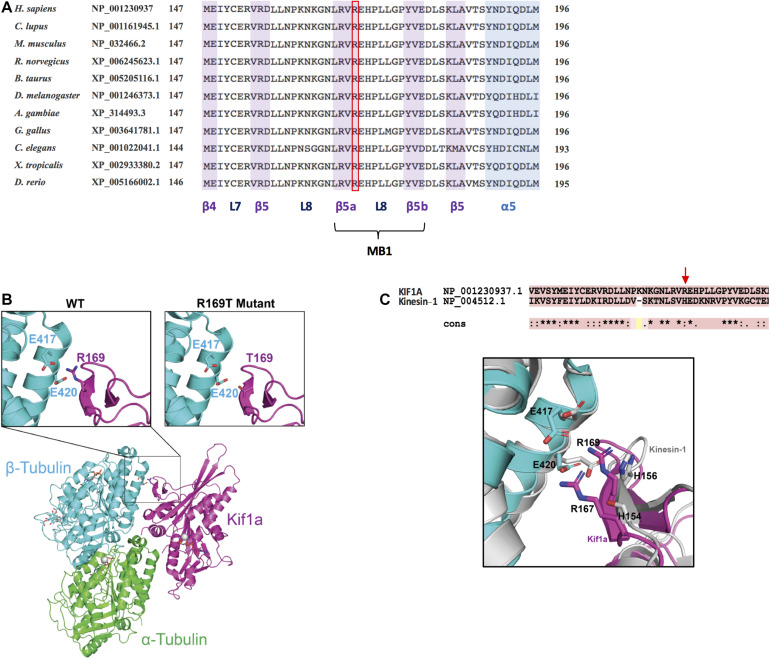
Conservation analysis and structural modeling of the R169T variant. **(A)** Multiple-sequence alignment showing the conservation of R169 residue in KIF1A across evolution. The alignment has been performed using HomoloGene (https://www.ncbi.nlm.nih.gov/homologene). L7, loop 7; L8, loop 8; MB1, microtubule-binding region 1. **(B)** Schematic representation of KIF1A bound to the alpha/beta tubular heterodimer. Insets show close-up views of (left) the electrostatic interaction mediated by KIF1A R169 with β-tubulin residues E417 and E420 and (right) the loss of electrostatic interaction caused by KIF1A R169T mutation. α-Tubulin is shown in green, β-tubulin in cyan, and KIF1A in pink. **(C)** Superposed structure of microtubule-bound Kinesin-1 (PDB: 3J8Y) onto microtubule-bound KIF1A (PDB: 2HXF).

The KIF1A kinesin motor domain is composed of a single globular catalytic core (1–361 aa) that contains domains for both ATPase activity and microtubule binding. The amino acid R169 is highly conserved throughout evolution, and it is located in the β5-loop 8 corresponding to one of the three microtubule-binding regions (together with loop 11 and loop 12) (Figure 3A; Kikkawa et al., 2000; Scarabelli et al., 2015). The variant R169T eliminates an arginine that has a positively charged side chain and introduces a threonine that has a polar uncharged side chain. As shown in Figure 3B, R169 is involved in electrostatic interactions with E417 and E420 of β-tubulin. Mutating R169T likely weakens the electrostatic interaction between the KIF1A protein and β-tubulin, suggesting a reduced KIF1A interaction with microtubules, which might ultimately cause the observed reduction in the microtubule-stimulated ATPase activity.

To deepen into the contribution of the β5-loop 8 for microtubule binding in KIF1A, we superposed the structure of the microtubule-bound KIF1A (PDB: 2HXF) onto microtubule-bound Kinesin-1 (PDB: 3J8Y). The substitution of H156A mildly affected K_*m*_ and K_cat_ (1.62 ± 0.43 and 1.12 ± 0.02, respectively) (Woehlke et al., 1997). On the contrary, the substitution R169T on KIF1A strongly affects K_*m*_ and K_cat_ (Figure 2F). R169 and R167 into the β5-loop 8 of KIF1A make more interactions with microtubules as compared to the equivalent residues H156 and S154 in Kinesin-1 (Figure 3C). In KIF1A, both R169 and R167 make hydrogen bonding interactions with tubulin residues, E420 and E417. However, equivalent residues in Kinesin-1, H156, and S154 are not within hydrogen bonding distance to E420 and E417 tubulin amino acids (Figure 3C). In addition, a 67.4-Å^2^ surface area of R169 is contributed to the total surface area buried upon KIFA–tubulin complex formation, which is 5% of the total area buried (1,324 Å^2^) at the binding interface of β-tubulin and KIF1A. Nevertheless, only 23.3 Å^2^ is buried in Kinesin-1, which is 1.8% of the total area buried (1,271 Å^2^) at the interface between β-tubulin and kinesin-1 (not shown). Together, these results show that the contribution of H156 for microtubule binding in Kinesin-1 is relatively minor as compared to that of R169 in KIF1A, likely explaining why mutating R169 in KIF1A has a stronger effect as compared to that of H156 on Kinesin-1 (Woehlke et al., 1997).

## Discussion

*KIF1A* encodes a neuron-specific motor protein that plays an important role in anterograde axonal transport of synaptic vesicle precursors. Pathogenic variants in the *KIF1A* gene have been associated with a wide spectrum of neurological phenotypes (Nemani et al., 2020) ranging from recessive hereditary sensory neuropathy-type IIC (HSNIIC) to autosomal recessive or dominant hereditary spastic paraplegia 30 (SPG30) and to the most severe NESCAV syndrome (NESCAVS). There are approximately more than 80 variants located in the motor domain and associated with KIF1A-related disorders (Kaur et al., 2020). The differences in disease severity between NESCAVS, with a severe neurological involvement, and the relative mild HSNIIC and both recessive and dominant SPG30 forms is thought to be due to the effect of the variant on protein transport by the KIF1A protein.

It is hypothesized that mild transport defects would be responsible for the hereditary spastic paraparesis phenotype, whereas severe defects in transport would be responsible for the NESCAVS phenotype (Pennings et al., 2020). However, a recent study has shown that some missense variants associated with SPG30 (V8M, A225V, and R350G) located in the motor domain produce a hyperactivation of KIF1A motor activity *in vitro* (10–20-fold higher than the WT), resulting in an increased anterograde transport of synaptic vesicles in *Caenorhabditis elegans* (Chiba et al., 2019).

Therefore, the functional analysis of KIF1A variants on the motility of the protein is crucial to understanding the effect of the variant on the phenotype. Even variants affecting the same amino acid can cause different phenotypes. Variants G102S, R167H, and R350W are found in dominant SPG while variants G102D and R167C cause NESCAVS and R350G recessive SPG.

Here, we report a novel *de novo* missense variant (R169T) located in the motor domain of the KIF1A protein. The patient presents the typical features of NESCAV syndrome, severe developmental delay, ataxia, spasticity, progressive cerebellar atrophy, peripheral neuropathy, and optic bilateral atrophy (Table 1 and Figure 1). First, we showed that the full-length KIF1A WT and R169T mutants were expressed in similar levels in human neuronal cells and with identical electrophoretic mobility, thereby suggesting no major alterations in protein stability. However, we cannot exclude the possibility that the endogenous protein in the patient might be unstable. In fact, a computational analysis of 22 KIF1A and 18 KIF5A disease-causing variants located in the motor domain showed changes of the folding free energy (ΔΔG) of the R169K variant in KIF1A, indicating that it may decrease protein stability (ΔΔG = 0.948) (Mahase et al., 2020). However, the R169T substitution was not tested. The expression and purification of the motor domain constructs in *E. coli* used in our study worked at fairly similar efficiencies, indicating that the R169T mutation may not cause a global misfolding of the motor domain.

To assess the impact of the R169T variant on KIF1A functionality, we expressed and purified the motor domain (3–362 aa) as a 6× His fusion protein from E. coli. We have particularly chosen this minimal, monomeric construct, as it is known that regions following the motor domain are involved in the auto-regulation of the motor activity and the dimeric form is kept inactive by auto-inhibitory mechanisms. Using an ATPase assay, we could demonstrate that the R169T-mutated motor domain has an impaired ATPase activity compared with the WT motor domain (Figure 2D). Consequently, the K_cat_ value is about 8-fold decreased in the R169T mutant. Furthermore, the strong increase in the K_*m*_ value for microtubules suggests that the impaired activity of the mutant motor domain R169T is caused by a reduced stimulation of the ATPase activity by microtubules. In support of this hypothesis, the 3D modeling of the R169T variant predicts a disruption in the binding of KIF1A to β-tubulin, thereby suggesting that the lack of ATPase activity and motility is probably a consequence of weakened binding of R169T to microtubules.

Other missense variants reported in the motor domain and associated with NESCAV syndrome (T99M, R216C, and E253K) show no motility in gliding assays, and in the case of the variant E253K, a dominant negative effect has been proven (Esmaeeli Nieh et al., 2015). Dimeric kinesins like KIF1A require the activity of both motor domains for the processive movement along microtubules. It is therefore tempting to speculate that like in the case of E235K, the heterozygous R169T variant, which completely inhibits the ATPase activity of KIF1A, might exert a dominant negative effect.

Several variants have been reported to be located in regions involved in microtubule binding (loop 8 and loop 12). Among them, the *de novo* missense variant R167C is located within the same microtubule binding region 1 (β5-loop 8) as the R169T variant reported here. 3D modeling of the R167C missense variant shows that it weakens the binding to microtubules, reducing the velocity and processivity of the kinesin (Lee et al., 2015; Scarabelli et al., 2015). Another variant, R307Q, located in the microtubule binding region 3 [α4-loop 12 (K-loop)-α5] reduces the positive charge of the K-loop decreasing the affinity of the motor domain to the microtubules (Hotchkiss et al., 2016). Recently, Kaur et al. reported a *de novo* missense variant (P305L) located in loop 12 of the motor domain and showed that the protein with the P305L variant accumulates in the cell body and that binding to microtubules was severely impaired (Kaur et al., 2020).

In conclusion, we describe here the effect on the KIF1A motor domain of a novel *de novo* missense variant associated with severe developmental delay, spastic paraparesis, motor sensory neuropathy, bilateral optic nerve atrophy, progressive cerebellar atrophy, epilepsy, ataxia, and hypotonia. We show that the R169T variant present in our patient strongly reduces ATPase activity and therefore protein motility, probably due to defects in microtubule binding as shown in structural modeling. The characterization of the molecular effect of the R169T variant on the KIF1A protein together with the presence of the typical clinical features indicates its causal pathogenic effect.

## Data Availability Statement

The datasets presented in this study can be found in online repositories. The names of the repository/repositories and accession number(s) can be found below: ENA, PRJEB41088.

## Ethics Statement

The studies involving human participants were reviewed and approved by the Institutional Ethics Committee of Institut d’Investigació i Innovació Parc Taulí I3PT (CEIC 2019/514). Written informed consent to participate in this study was provided by the participants’ legal guardian/next of kin. Written informed consent was obtained from the individual(s), and minor(s)’ legal guardian/next of kin, for the publication of any potentially identifiable images or data included in this article.

## Author Contributions

CA, SH, MM, MS, AS, and AR: design of the work. CA, SH, MM, AJ, EG, MG, MS, AS, and AR: data collection. CA, SH, AJ, MM, MS, AS, and AR: data analysis and interpretation. CA, SH, AJ, MS, AS, and AR: manuscript preparation. All authors are contributing to manuscript revision.

## Conflict of Interest

The authors declare that the research was conducted in the absence of any commercial or financial relationships that could be construed as a potential conflict of interest.
