# Supplementary material for: The Novel KIF1A Missense Variant (R169T) Strongly Reduces Microtubule Stimulated ATPase Activity and Is Associated With NESCAV Syndrome
Source: Front Neurosci. 2021 May 26;15:618098. doi: 10.3389/fnins.2021.618098 (PMC8187576; doi:10.3389/fnins.2021.618098)
Supplement: Supplementary file 1 [file Data_Sheet_1.docx]

Supplementary Material

# Supplementary Figures

##

**B)**

**A)**


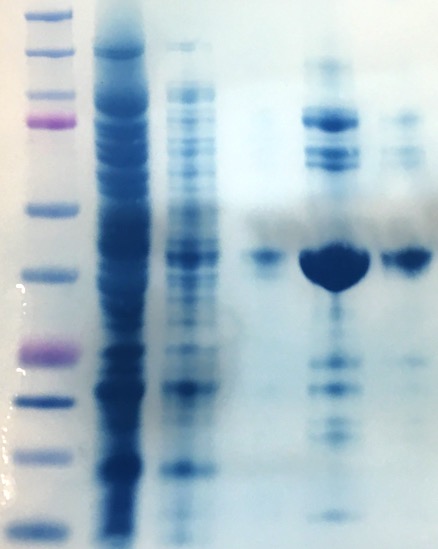


KIF1A (3-362)^R169T^

150

100

75

50

37

25

15

20

kDa

250

10

Wash 1

Wash 2

Elution 1

Elution 2

Lysis


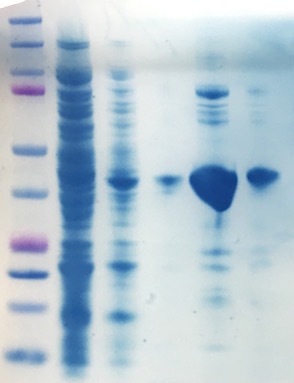


150

100

75

50

37

25

15

20

kDa

250

10

Wash 1

Wash 2

Elution 1

Elution 2

KIF1A (3-362)^WT^

Lysis

**C)**

**Figure 1: Detection and purification of His tagged KIF1A motor domain.** **A)** Expression of KIF1A motor domain WT and R169T after 1mM IPTG induction at 18 °C overnight of BL21 cell culture. 10 µl of the cell pellet resuspended with Laemmli Buffer 2x was loaded. Red arrowheads indicate the recombinant protein corresponding to KIF1A motor domain. **B)** Western blot that shows the specific expression of the recombinant motor domain of KIF1A that carry a six-histidine tail. A penta-his antibody (1/1000) was used to detect the recombinant proteins. **C)** Steps on the purification of KIF1A WT and R169T motor domains. Red arrowheads indicate the purified recombinant proteins.

# Supplementary Tables

**Table 1.** Site directed mutagenesis primers.

| **Primer name** | **Primer sequence (5’🡪 3’)** | **Length** | **Tm (⁰C)** |
| --- | --- | --- | --- |
| KIF1A_R169T_F | GGGTGCTCCGTCACGCGAAGGTTGCCC | 27 | 84,8 |
| KIF1A_R169T_R | GGGCAACCTTCGCGTGACGGAGCACCC | 27 | 84,8 |

| **Gene** | **Genomic coordinates (hg19)** | **Nucleotide change** | **Amino acid change** | **Variant type** | **ACMG classification** | **Gene function** |
| --- | --- | --- | --- | --- | --- | --- |
| *KIF1A* | Chr2:241725854C>G | NM_001244008.2:c.506G>C | NP_001230937.1:p.(Arg169Thr) | Missense | Likely pathogenic | KIF1A belongs to the kinesin-3 family of kinesins and is involved in fast anterograde transport of synaptic vesicle precursors along microtubules. |
| *RCC2* | Chr1:17748698C>A | NM_001136204.3:c.744+1G>T | - | Splicing | Likely pathogenic | *RCC2* encodes for a multifunctional protein that regulates the activity of small GTPases and is required for normal progress of the cell cycle during interphase and mitosis. |
| *ZNF2* | Chr2:95847733G>A | NM_021088.4:c.1160G>A | NP_066574.2 :p.(Arg387Gln) | Missense | Variant of uncertain significance | ZNF2 may be involved in DNA binding and transcriptional regulation. |

**Table 2.** Variants identified after filtering WES trio data for a *de novo* pattern of inheritance.
